# Psychological antecedents of vaccine inequity: keys to improve the rates of vaccination

**DOI:** 10.1186/s42506-024-00175-7

**Published:** 2024-12-04

**Authors:** Mohamed F. Hussein, Sarah A. Ibrahim, Suzan Abdel-Rahman, Abdelhamid Elshabrawy, Haqema A. A. Nasr, Saja Yazbek, Abdul Jabbar, Cinaria T. Albadri, Mariam Alsanafi, Narjiss Aji, Naglaa Youssef, Hammad M. Hammad, Fatimah S. A. Abdullah, Ehab Elrewany, Mohamed M. Tahoun, Mahmoud Tolba, Mohamed K. Abo Salama, Ramy M. Ghazy

**Affiliations:** 1 Occupational Health and Industrial Medicine Department, High Institute of Public Health, Alexandria University, Alexandria, Egypt; 2 Biostatistics and Demography Department, Faculty of Graduate Studies for Statistical Research, Cairo University, Cairo, Egypt; 3 Parasitology and Immunity Department, Faculty of Medicine and Health Sciences, Amran University, Sanaa, Yemen; 4 Faculty of Public Health, Lebanese University, Beirut, Lebanon; 5 Department of Veterinary Medicine, Faculty of Veterinary Science, University of Veterinary and Animal Sciences, Lahore (Punjab), 54600 Pakistan; 6 Dnipro Medical Institute of Traditional and Non-Traditional Medicine, Dublin, Ireland; 7 Department of Pharmacy Practice, Faculty of Pharmacy, Kuwait University, Kuwait City, Kuwait; 8 Faculty of Medicine and Pharmacy of Rabat, Rabat, Morocco; 9 Department of Medical-Surgical Nursing, College of Nursing, Princess Nourah Bint Abdulrahman University, Riyadh, Saudi Arabia; 10 Al-Mana General Hospital, Khartoum, Sudan; 11 Internal Medicine Department, Faculty of Medicine, Alexandria University, Alexandria, Egypt; 12 Tropical Health Department, High Institute of Public Health, Alexandria University, Alexandria, Egypt; 13 Department of Epidemiology, High Institute of Public Health, Alexandria University, Alexandria, Egypt; 14 Clinical Research Department, Ministry of Health and Population, Faiyum, Egypt; 15 Faculty of Medicine, Al-Azhar University, Cairo, Egypt

**Keywords:** COVID-19 vaccine, The Middle East and North Africa, 5C scale, Inequity of vaccines, Social determinants of health, Universal health coverage

## Abstract

**Background:**

The World Health Organization (WHO) stresses the importance of worldwide vaccine coverage of coronavirus-19 (COVID-19) vaccination. This study fills a critical gap in the literature by providing empirical evidence on the factors influencing COVID-19 vaccine hesitancy and inequity in the Middle East and North Africa (MENA) region. This study investigated the determinants of psychological antecedents and other factors behind COVID-19 vaccination and their role in vaccine coverage in MENA.

**Methods:**

An anonymous online cross-sectional survey was conducted in 11 MENA countries (Egypt, Sudan, Kuwait, Saudi Arabia, Morocco, Iraq, Yemen, Lebanon, Libya, Afghanistan, and Pakistan). The minimum required sample size from each country was 307, which was increased to 330 to accommodate a non-response rate of 7%. A multilevel logistic regression model was used to capture the clustering of observations in each country and estimate the explanatory variables’ effects on each item of the 5C components of the psychological antecedents scale namely (confidence, constraints, complacency, calculation, and collective responsibility).

**Results:**

The total number of respondents was 3630, 40.5% of them were between the ages of 18 and 25 years, 61.1% were females, 54.0% completed university education, 55.8% were unmarried, 19.5% had chronic diseases, 43.7% reported a previous COVID-19 and 42.4% had relatives who died from COVID-19. Much of the variation in the log of the odds in each item of the 5Cs was due to heterogeneity between different countries (intraclass correlation > 0.05). Therefore, this variability confirms the various effects of psychological antecedents on vaccination coverage, stimulating vaccination inequity among them. Increasing confidence in vaccines and collective responsibility towards relatives and the community is related to increasing acceptance of the COVID-19 vaccine. The reduction in complacency, calculations, and constraints was found to be associated with acceptance of the COVID-19 vaccine.

**Conclusions:**

This study is novel in shedding light on the importance of psychological determinants as hidden causes of vaccine inequities by using a multilevel logistic regression model for COVID-19 vaccination intention. The findings suggest that targeted interventions addressing socio-demographic factors, psychological antecedents, and accessibility barriers are essential to mitigate vaccine inequity and improve vaccination rates.

**Supplementary Information:**

The online version contains supplementary material available at 10.1186/s42506-024-00175-7.

## Introduction

High mortality and morbidity rates have overwhelmed the world since the emergence of coronavirus disease 2019 (COVID-19). This pattern of infection and mortality varied across countries [1]. As of 15 June 2024, more than 775 million confirmed cases of COVID-19 and more than 7 million deaths due to COVID-19 have been reported worldwide [2]. The pandemic has negatively affected the socio-economic and psychological state of the population while exhausting the healthcare workers and healthcare system [3].

Consequently, efficient preventive measures are necessary, with extensive efforts focused on the development of effective and safe vaccines against COVID-19 [4]. Vaccines are a powerful and affordable tool. They not only prevent serious illnesses, disabilities, and deaths from diseases they target but also lessen the impact of outbreaks like COVID-19 in countries with limited healthcare resources. This makes vaccination crucial for protecting public health, especially in developing countries [5]. Although vaccines have spread widely, many cases are discovered every day [2]. Most countries in the Middle East and North Africa (MENA) are still in the community transmission phase, which is a state of inability to relate confirmed cases through transmission chains in many cases, resulting in a high burden on healthcare facilities and the economy [6].

The emergence of new variants and subvariants of COVID-19 continues to pose a significant global threat. These variants have the potential to increase infection rates and challenge the effectiveness of current vaccines. This ongoing virus evolution highlights the importance of continued research and vaccine development efforts [7].

To control the pandemic and prevent the emergence of new variants, achieving herd immunity through widespread vaccination is crucial. This typically requires vaccinating 60–80% of the population. Unfortunately, many countries are far from reaching these targets. This underscores the urgent need for equitable vaccine access [8].

One of the main reasons for the continuation of the pandemic is the low vaccination rate due to vaccine hesitancy. In 2019, even before the start of the COVID-19 pandemic, vaccination hesitancy was one of the top ten threats to global health and has been defined as “reluctance or refusal to vaccinate despite the availability of vaccines” [9]. Vaccine-hesitant individuals are heterogeneous groups with different degrees of hesitancy regarding specific vaccines or vaccinations. They may accept all vaccines but remain concerned about them; some may refuse or delay some vaccines but accept others, while others may refuse all vaccines [10].

The World Health Organization (WHO) stressed the importance of vaccine equity to achieve acceptable worldwide distribution and coverage of COVID-19 vaccination [11]. Although many doses of COVID-19 vaccines have been administered worldwide and many people have received at least one dose of vaccination, the actual situation is different. High-income countries have exceeded 90% vaccine coverage, while other countries have low vaccination coverage. Even within each country, vaccination coverage varies from area to area and from one group of people to another [12].

Vaccine hesitancy and lack of access are two main hurdles to achieving widespread vaccine equity. Physical barriers like cost, accessibility, and supply chain constraints can prevent people from getting vaccinated. In addition to these practical challenges, psychological factors also play a role. People's beliefs, attitudes, and feelings about vaccines can influence their willingness to get vaccinated. Social drivers like what others around them are doing and their motivations for getting vaccinated can also affect decisions [13]. In a scoping review study, Bergen et al. [14] stated that COVID-19 vaccine inequity is mostly related to age, race, and sex. Higher vaccination coverage was found among older people, whereas males tend to take vaccinations slightly more than females.

Generally, an individual’s acceptance of receiving a vaccine is affected by various factors, such as misleading and huge amounts of infodemics, fear of undesired side effects, lack of trust in its effectiveness, lack of confidence in health system authorities and health care workers, and lack of reports on the safety and efficacy of COVID-19 vaccines [15]. Several theoretical models have been developed to investigate the underlying causes of vaccine hesitancy. The 5C scale is a helpful tool for understanding the reasons behind vaccine hesitancy. It focuses on five key psychological factors influencing a person's decision to get vaccinated. This scale includes confidence (efficacy and safety of the vaccine), complacency (no essential risks have been found from the disease), constraints (vaccination logistics that enable or hinder vaccination), calculation (indicating the availability of medical information about the vaccination), and collective responsibility (assessing the public readiness and awareness that one's vaccination safeguards others). By analyzing these five aspects, the 5C scale helps healthcare professionals and researchers identify the specific reasons behind vaccine hesitancy and develop targeted strategies to address them [16]. The model revealed that uncertainty about the effectiveness, side effects, and effective duration of the COVID-19 vaccine were among the most important factors that could increase vaccine hesitancy [17].

**Fig. 1:**
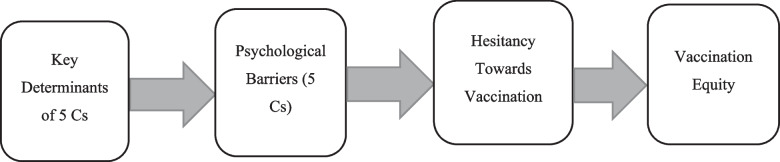
The pathways to vaccine equity, in Middle East and North African countries

Figure 1 shows the pathways to vaccine equity. There are important determinants of the 5Cs, such as the demographic and socioeconomic characteristics of the respondents. Thus, the 5Cs, as explained earlier, are the psychological factors that precede vaccination, including personal beliefs, attitudes, and perceptions, which can directly influence vaccine hesitancy and, in turn, vaccination rates, as manifested by disparities among countries. However, socioeconomic variables may contribute indirectly to vaccination inequalities in the countries of the MENA.

In the MENA region, approximately 64% of the population has received two initial doses of COVID-19 vaccines. In low-income countries, only 32.8% of people received at least one vaccination dose. In the MENA region, the percentage of those who completed the initial protocol varies from one country to another (78% in Kuwait, 70% in Saudi Arabia, 63% in Morocco, 60% in Pakistan, 52% in Tunisia, 44.7% in Afghanistan, 44% in Lebanon, 38% in Egypt, 29% in Sudan, 17.9% in Iraq, and 2.4% in Yemen) till 31 December 2023 [18]. Exploring the determinants of COVID-19 vaccine hesitancy and the desire to receive it among the general population in MENA has not been completely detected. Therefore, it was essential to conduct this large-scale survey to identify the factors that affect the hesitancy to receive the COVID-19 vaccine using the 5C scale. While some studies have focused on the effect of 5Cs on COVID-19 vaccination hesitancy [17], few studies have examined the determinants of the 5Cs, which are the main drivers of vaccination hesitancy and equity, particularly in the MENA region [1, 19].

This study aimed to identify the psychological antecedents of vaccination and the potential correlation between 5Cs and vaccine coverage. It also aimed to identify how individual beliefs, attitudes, and perceptions can influence vaccination coverage and how socioeconomic factors impact vaccination inequity in MENA countries 2 years after starting vaccination programs.

## Methods

### Study design and setting

An anonymous online-based cross-sectional survey was conducted from 20th September to 9th December 2022 in 11 countries of the MENA (Egypt, Iraq, Kuwait, Lebanon, Libya, Morocco, Pakistan, Afghanistan, Sudan, Saudi Arabia, and Yemen) by spreading the questionnaire via social media (Facebook, Twitter, WhatsApp, Telegram, etc.).

### Study population

Participants were eligible for participation in this study if they were older than 18 years, could read Arabic or English, could access the Internet through a computer or smartphone to answer the electronic questionnaire, and were residents in the MENA region during the COVID-19 pandemic.

### Sampling method and sample size

Convenience and snowball sampling methods were used to recruit participants with the required sample sizes. Researchers used a combination of convenience and snowball sampling to recruit participants. First, collaborators recruited people who were easy to reach and from different backgrounds to ensure a diverse sample. Then, these participants helped recruit others from their social networks until we had enough participants from each country. According to Maas and Hox [20], 330 observations per country were a sufficient sample size for multilevel logistic regression models. We included 11 countries from Africa and Asia, with 330 observations per country, resulting in 3630 observations. We utilized G. Power software (statistical test: logistic model) to calculate the necessary sample size for the countries included in the study. The minimum required sample size from each country was calculated with an error rate (α) of 0.05, a power of 80%, and a probability of having any of the 5Cs (p1) at 0.4, considering the different prevalences of the 5Cs detected in the sample. The prevalence of having any of the constraints in our data set ranges between 5 and 33%. To cover the whole range, we used 40% (P1 = 0.4) as an assumption of having any of the constraints. Additionally, we accounted for the variance of one of the explanatory variables explained by the other independent variables (0.3). This moderate level of correlation (0.3) is often used in sample size calculations when precise information about the correlations is not available, but some degree of association is expected [21]. The non-response rates between 5 and 10% are typical for many surveys and do not usually compromise the validity of the results if managed properly [22].

Consequently, the minimum required sample size from each country was 307, which was increased to 330 to accommodate a non-response rate of 7% [21]. Equal sample sizes ensure that each country's data contributes equally to the overall analysis, providing consistent statistical power across countries. This uniformity can help detect meaningful differences or relationships among the 5Cs (e.g., confidence, convenience, complacency, calculation, and collective responsibility) across all countries, assuming the effect sizes are similar.

### The data collection tool

The researchers designed an anonymous electronic questionnaire using Google Forms that was connected directly to an Excel sheet for automatic data transfer and analysis. To examine the practicality and accessibility of the online questionnaire, a pilot study was conducted in which each researcher sent the questionnaire to at least three individuals. In total, 185 people participated in the pilot study, representing about 5.1% of our total sample (3630 participants). The results of the pilot study indicated that the questionnaire takes 5–10 min to be fully answered and that few sentences required rephrasing for clarity. Participants were also restricted from submitting multiple responses to ensure data accuracy. To address the potential impact of language and cultural nuances on participant responses, we utilized a validated questionnaire available in both Arabic and English. The questionnaire was revised based on feedback from the pilot study, and participants were encouraged to contact the research team for clarification. These strategies aimed to ensure that the questionnaire was understood and answered accurately by participants from all cultural backgrounds.

The questionnaire consisted of two parts:

The first part included socio-demographic characteristics (participants’ age, sex, nationality, residence, marital status, occupation, and education), medical history, history of previous COVID-19 infection, COVID-19 vaccination status, and history of death from COVID-19 infection among participants’ relatives or friends. The occupations were classified into four major categories: high-skilled (non-manual) occupations include legislators, senior officials, managers, professionals, technicians, and associate professionals; low-skilled (non-manual) occupations include clerks, service workers, and market sales workers; skilled manual occupations include skilled agriculture and fishery workers, craft and related trades workers, plant and machine operators, and assemblers; others include elementary workers, nonworking participants, and students [23].

The second part involved vaccine hesitancy questions, which were investigated using a 15-item tool developed from a “5C model” of psychological antecedents to vaccination. The current research used the validated Arabic and English versions of the 5C scale, a well-established instrument for assessing the psychological antecedents of vaccination. The 5C scale has been validated in previous studies [16, 24]. Using these validated versions of the 5C scale guaranteed the reliability and validity of the data collected in the current study. The psychological antecedents of vaccination of 5C include five domains: confidence, complacency, constraints, calculation, and collective responsibility [16]. Participants responded to each of the 15 items using a 7-point Likert scale ranging from 1 (strongly disagree) to 7 (strongly agree). The scores of the three questions representing each domain were calculated, and the mean scores of each domain were classified as yes and no. In general, a sense of confidence and collective responsibility are associated with a positive attitude toward vaccination, whereas increased complacency, constraints, and calculation are associated with vaccine hesitancy.

The 5C subscales were classified according to the cutoff values according to *No* for confidence (< 5.7 vs. ≥ 5.7), complacency (< 4.7 vs. ≥ 4.7), and constraints (< 6.0 vs. 6.0), evaluation of the calculation of COVID-19 (< 6.3 vs. ≥ 6.3), and collective responsibility (< 6.2 vs. ≥ 6.2) [25]. The questionnaire was available in Arabic and English (Supplementary material S1). The questionnaire showed an acceptable level of internal consistency, where its Cronbach’s alpha was 0.764 (0.75–0.78).

### Variables

The outcome variable of this study was the 5C scale: confidence, constraints, complacency, collective responsibility, and calculations. The 5C scale was coded as a binary variable based on a threshold available in the literature [25]. This study examined the determinants of the 5C scale by applying a multilevel logistic regression model including 11 countries in the MENA region.

The independent variables were selected at two levels: country and individual. Age, sex, educational level, social status, occupation of the respondents, suffering from a chronic disease, previous COVID-19 infection, and experiencing death from COVID-19 within the family were at the individual level while the continent was added at the country level.

### Statistical analysis

Statistical analysis was carried out using the R package called lme4 [26]. Counts and percentages for categorical variables were applied. The chi-squared (*χ*2) test of independence was carried out to check the relationship between the dependent variables (confidence, complacency, constraints, assessing calculations, and collective responsibility) and independent variables such as age, sex, educational level, occupation, suffering from a chronic disease, and previous COVID-19 infection. Statistical significance was set at *P* < 0.05. A point-biserial correlation was used to measure the strength and direction of the association between one continuous variable and one dichotomous variable. This is a special case of Pearson’s product-moment correlation, which is applied when there are two continuous variables; in this case, one of the variables is measured on a dichotomous scale.

#### Data analysis and model

A multilevel logistic regression model was employed to account for the clustering of observations within each country. Additionally, it assesses the impact of explanatory variables on each component of the 5C psychological factors across 11 countries in the MENA Region. Nevertheless, neglecting the hierarchical structure of the data and assuming independence among observations at the first level can lead to inaccurate model estimates. If the intraclass correlation exceeds 0.05, failing to apply a multilevel approach can bias parameter estimates and standard errors.

The single-level logistic regression model was improved by adding random slopes and intercepts to the model. The analysis was performed in consecutive steps before estimating the final model [27]. First, the null model with only the intercept was estimated with a fixed parameter, and then we added the random-effects parameter. The log-likelihood ratio test and intraclass correlation (ICC) were used to evaluate the multilevel model with a random intercept. The final model takes the following form:

1 $$Logit(\frac{{Y}_{ij}}{1-{Y}_{ij}})={\beta }_{00}+\left({\beta }_{10}+{U}_{1j}\right){X}_{ij}+{\beta }_{01}{X}_{j}+{U}_{0j}$$

Where the proportion ($P({Y}_{ij}=1$) to (1-$P({Y}_{ij}=1))$ was the odds of each of the 5C scale, ${\beta }_{00}$ was the fixed intercept (the average log of odds across the countries), ${U}_{0j}$ was the deviation of the country intercept (the random effect of the intercept). It was assumed that the random effect followed a normal distribution with variance ${\sigma }_{uo}^{2}$. Moreover, we added the random effect part to the slope of the independent variables. ${X}_{ij}$ was the value of the independent variable (first-level variables), ${\beta }_{10}$ was the fixed slope, ${U}_{1j}$ was the country-specific slope deviation for the selected variables, ${X}_{j}$ was the value of country-level variables, ${\beta }_{01}$ was the fixed slope of country-level variables.

To assess the goodness of fit of the models, we used the Akaike information criterion (AIC), the Bayesian information criterion (BIC), and the likelihood ratio test; the lower the indices, the better the model [28]. Additionally, the Likelihood Ratio Test (LRT) was used to compare the fit of two nested models—one with a random slope and one without. The results of the LRT reveal whether including a random slope significantly improves the model’s fit compared to a simpler model. If the LRT yields a significant p-value, it indicates that the model with the random slope provides a substantially better fit to the data, justifying the inclusion of the random slope to account for variability in the effect of the independent variable across different levels (e.g., countries).

##### Multi-level logistic regression model

In the following section, we present the results of the null model first with a fixed part of the intercept and then with the addition of the random effects part. The improvement in the model was checked after adding the random-effects part using the likelihood ratio test.

The odds of being confident, complacent, constrained, performing calculations, and having collective responsibility were 0.32, 0.30, 0.05, 0.36, and 0.25 in different countries. We conducted a null model using only the fixed part. The likelihood ratio test indicated a significant difference between the two models (${\chi }^{2}$ = 398.5, 63.6, 34.5, 343.5, and 250.8, at *p* < 0.001). Additionally, intraclass correlations differed across the 5C scale. However, all of them exceeded 0.05 except complacency (0.05),^1^ indicating that the single-level model was not appropriate for estimating clustered data in each country. Hence, we added a random effect part to the intercept, assuming a multilevel structure of the model. The intraclass correlation coefficient (ICC) values indicate varying degrees of country-level influence on the 5C components. For “confidence” (ICC = 0.22) and “constraints” (ICC = 0.10), there is moderate variability between countries, suggesting that while country-specific factors do play a role, a substantial amount of variance occurs within countries. In contrast, “calculation” (ICC = 0.38) and “collective responsibility” (ICC = 0.33) show higher ICC values, reflecting significant country-level variability, indicating that differences between countries are notably influential for these components. Conversely, “complacency” (ICC = 0.05) has a low ICC, implying that most of the variability is within countries rather than between them. These findings underscore the varying impact of country-level factors across different psychological components of the 5Cs.

The variance of the random intercept presents the average squared deviation of the intercepts from the overall mean intercept (the fixed intercept). A higher variance indicates greater variability in intercepts among countries. The table also shows that the variances of the random effects part of the intercept (σ^2^_00_) for confidence, complacency, constraints, calculations, and collective responsibility are 0.91. 0.16, 0.36, 2, and 1.64, respectively, indicating high variability in the calculation and collective responsibility model intercepts. Besides, the standard deviations are the square roots of the variances, indicating how much the intercepts vary across different countries (0.95, 0.4, 0.6, 1.4, and 1.2, respectively) (Table 1). The variations among the odds of the 5Cs in the 11 countries under study were presented in the Supplementary material S2: (Figs. A1–A5)].

**Table 1:** Null model results after adding random effects to the intercept, the Middle East and North African countries, 2022 (*N* = 3630)

	**Confidence**	**Complacency**	**Constraints**	**Calculation**	**Collective**
*Predictors*	*Intercept and odds ratios*	*Intercept and odds ratios*	*Intercept and odds ratios*	*Intercept and odds ratios*	*Intercept and odds ratios*
(Intercept)	(− 1.1435, OR = 0.32 ^***^, Std.Error = 0.9, 95% CI 0.18 – 0.57)	(− 1.2266, OR = 0.30^***^, Std.Error = 0.04,95% CI 0.23 – 0.38)	(− 2.9893, OR = 0.05^***^, Std.Error = 0.01,95% CI: 0.03 – 0.07)	(− 0.9723, OR = 0.36^*^, Std.Error = 0.17,95% CI: 0.16 – 0.85)	(− 1.3694, OR = 0.25 ^***^, Std.Error = 0.1, 95% CI 0.12 – 0.55)
**Random effects**	**Random effects**	**Random effects**	**Random effects**	**Random effects**	**Random effects**
σ^2^_00_	0.91 _Countries_	0.16 _Countries_	0.36 _Countries_	2.00 _Countries_	1.64 _Countries_
ICC	0.22	0.05	0.10	0.38	0.33
No.	11 _Countries_	11 _Countries_	11 _Countries_	11 _Countries_	11 _Countries_
Observations	3630	3630	3630	3630	3630

*OR* Odds Ratio, *CI* Confidence Interval, *Std error* Standard error

^*^P < 0.05,^**^P < 0.01, ^***^P < 0.001

After accepting the assumption of heterogeneity of the logit of odds across different countries, we conducted five models with random intercepts and added explanatory variables. The significance of the random slope for each significant independent variable was tested using the likelihood ratio test for the nested models. Consequently, the random slope of the COVID-19 infection was added to the confidence model. In addition, we added a random slope of educational level to the complacency and constraint models. Finally, we added the COVID-19 death random slope to the calculation model. Therefore, adding the random slope for the variables mentioned above indicates that the effects of these variables differ between countries (level two).^2^

## Results

### Characteristics of the respondents

The total number of respondents was 3630, 40.5% were between the ages of 18 and 25 years, 61.1% were females, 54.0% completed a university education, 55.8% were unmarried, 38.0% worked in high-skilled occupations, and 72.7% were from Asian countries (Table 2).

**Table 2:** Distribution of respondents according to their socio-demographic characteristics, in the Middle East and North African countries, 2022 (*N* = 3630)

Characteristics	No. (%)
Age categories	
18– < 25 years	1470 (40.5)
25– < 35 years	1023 (28.2)
35– < 50 years	751 (20.7)
50–65 years	266 (7.3)
Above 65 years	120 (3.3)
Gender	
Female	2216 (61.1)
Male	1414 (38.9)
Level of education completed	
Primary education	52 (1.4)
Secondary education	729 (20.1)
University education	1961 (54.0)
Post-graduation	888 (24.5)
Social status	
Unmarried	2027 (55.8)
Married	1603 (44.2)
Occupation	
High-skilled (nonmanual)	1378 (38.0)
Low-skilled (nonmanual)	343 (9.5)
Skilled (manual)	85 (2.3)
Others	1824 (50.2)
Continent	
Asia	2640 (72.7)
Africa	990 (27.3)

About one-fifth of the respondents had chronic diseases (19.5%), 43.7% reported a previous infection with COVID-19, and 42.4% had relatives who died from COVID-19. Regarding the 5C scale, approximately one-quarter of the respondents were confident (27.4%), complacent (23.6%), had collective responsibility (25.4%); 33.1% performed calculations for receiving the COVID-19 vaccine, and 5.2% had constraints (Table 3).

**Table 3:** Distribution of respondents according to COVID-19 infection, COVID-19 vaccination, and the 5C scale, the Middle East and North African countries, 2022 (*N* = 3630)

Characteristics	No. (%)
Have chronic diseases	
Yes	707 (19.5)
No	2922 (80.5)
Have a previous COVID-19 infection	
No	1484 (40.9)
Yes	1586 (43.7)
I don't know	560 (15.4)
Have family members/relatives died from COVID-19?	
No	1785 (49.2)
Yes	1537 (42.4)
Maybe	308 (8.4)
Confidence	
Not confident	2636 (72.6)
Confident	994 (27.4)
Complacency	
Not complacent	2772 (76.4)
Complacent	858 (23.6)
Collective responsibility	
Lack of collective responsibility	2708 (74.6)
Having collective responsibility	922 (25.4)
Constraints	
Did not have constraints	3443 (94.9)
Had constraints	187 (5.1)
Calculation	
Did not perform calculation	2427 (66.9)
Perform a calculation	1203 (33.1)

### Explanatory variables associated with the dependent variables

Age was significantly related to confidence (*p* = 0.02), calculations (*p* < 0.001), and collective responsibility (*p* = 0.003). Education level had a statistically significant association with confidence (*p* = 0.005), constraints (*p* = 0.015), and calculations (*p* = 0.031). Social status had a significant association with calculations only (*p* = 0.026). Occupation status was significantly related to calculation (*p* = 0.043) and collective responsibility (*p* < 0.001). The association between chronic diseases and calculation and collective responsibilities was significant *p* < 0.001 and *p* = 0.013, respectively). Previous COVID-19 infection was significant with confidence only (*P* < 0.001). Having a dead relative from COVID-19 infection was significant with confidence (*p* = 0.001), calculations (*p* < 0.001), and collective responsibility (*p* = 0.02) (Table 4).

**Table 4:** The statistical relationship between the 5C scale and the socio-demographic characteristics of the respondents, experience related to COVID-19 infection, or COVID-19 vaccination, the Middle East and North African countries, 2022 (*N* = 3630)

Variables	Not confident	Confident	Not complacent	Complacent	No constraints	Having constraints	No calculations	Performing calculation	No collective responsibility	Collective responsibility
Age	*P*-value = 0.02^**^		*P*-value = 0.308		*P*-value = 0.074		*P*-value < 0.001^***^		*P*-value = 0.003^**^	
18– < 25 25– < 35 35– < 50 50–65 > 65	1050 (71.4) 761 (74.4) 566 (75.5) 178 (66.9) 81 (67.5)	420 (28.6) 262 (25.6) 185 (24.5) 88 (33.1) 39 (32.5)	1099 (74.8) 781 (76.3) 584 (77.8) 204 (76.7) 97 (80.8)	371 (25.2) 242 (23.7) 167 (22.2) 62 (23.3) 23 (19.2)	1382 (94.0) 966 (94.4) 721 (96.1) 255 (95.9) 118 (98.3)	88 (6.0) 57 (5.6) 30 (3.9) 11 (4.1) 2 (1.7)	998 (67.9) 686 (67.1) 481 (64.1) 162 (60.9) 99 (82.5)	472 (32.1) 337 (32.9) 270 (35.9) 104 (39.1) 21 (17.5)	1076 (73.2) 777 (76.0) 564 (75.2) 186 (69.9) 105 (87.5)	394 (26.8) 246 (24.0) 187 (24.8) 80 (30.1) 15 (12.5)
Gender	*P*-value < 0.001^***^		*P*-value = 0.023^**^		*P*-value < 0.001^***^		*P*-value = 0.003^**^		*P*-value = 0.001^***^	
Female Male	1672 (75.5) 964 (68.2)	544 (24.5) 450 (31.8)	1719 (77.6) 1048 (74.2)	497 (22.4) 366 (25.8)	2132 (96.2) 1311 (92.7)	84 (3.8) 103 (7.3)	1440 (65.0) 987 (69.8)	776 (35.0) 427 (30.2)	1612 (72.7) 1096 (77.5)	604 (27.3) 318 (22.5)
Educational level	*P*-value = 0.005^**^		*P*-value = 0.089		*P*-value = 0.015^**^		*P*-value = 0.031^**^		*P*-value = 0.122	
Primary education Secondary education University education Post-graduation	30 (57.7) 514 (70.5) 1417 (72.3) 675 (76.0)	22 (42.3) 215 (29.5) 544 (27.7) 213 (24.0)	37 (71.2) 548 (75.2) 1475 (75.2) 703 (79.2)	15 (28.8) 181 (24.8) 480 (24.8) 185 (20.8)	45 (86.5) 684 (93.8) 1864 (95.1) 850 (95.7)	7 (13.5) 45 (6.2) 97 (4.9) 38 (4.3)	37 (71.2) 455 (62.4) 1340 (68.3) 595 (67.0)	15 (28.8) 274 (37.6) 621 (31.7) 293 (33.0)	41 (78.8) 523 (71.7) 1489 (75.9) 655 (73.8)	11 (21.2) 206 (28.3) 472 (24.1) 233 (26.2)
Country	*P*-value < 0.001^***^		*P*-value < 0.001^***^		*P*-value < 0.001^***^		*P*-value < 0.0001^***^		*P*-value < 0.001^***^	
Egypt Sudan Kuwait Saudi Arabia Morocco Iraq Yemen Lebanon Libya AfghanistanPakistan	258 (78.2) 298 (90.3) 240 (72.7) 225 (68.2) 309 (93.6) 249 (75.5) 242 (73.3) 290 (87.9) 263 (79.7) 89 (27.0) 173 (52.4)	72 (21.8) 32 (9.7) 90 (27.3) 105 (31.8) 21 (6.4) 81 (24.5) 88 (26.7) 40 (12.1) 67 (20.3) 241 (73.0) 157 (47.6)	279 (85.1) 276 (84.7) 236 (71.7) 240 (73.2) 223 (67.6) 243 (73.9) 235 (71.2) 264 (80.0) 237 (72.0) 294 (89.1) 234 (70.9)	49 (14.9) 50 (15.3) 3 (28.3) 88 (26.8) 107 (32.4) 86 (26.1) 95 (28.8) 66 (20.0) 92 (28.0) 36 (10.9) 96 (29.1)	315 (95.5) 322 (97.6) 318 (96.4) 297 (90.0) 318 (96.4) 311 (94.2) 310 (93.9) 323 (97.9) 306 (92.7) 327 (99.1) 296 (89.7)	15 (4.5) 8 (2.4) 12 (3.6) 33 (10.0) 12 (3.6) 19 (5.8) 20 (6.1) 7 (2.1) 24 (7.3) 3 (0.9) 34 (10.3)	198 (60.0) 268 (81.2) 182 (55.2) 220 (66.7) 330 (100.0) 164 (49.7) 170 (51.5) 208 (63.0) 216 (65.5) 239 (72.4) 232 (70.3)	132 (40.0) 62 (18.8) 148 (44.8) 110 (33.3) 0 (0.0) 166 (50.3) 160 (48.5) 122 (37.0) 114 (34.5) 91 (27.6) 98 (29.7)	207 (62.7) 281 (85.2) 219 (66.4) 239 (72.4) 330 (100.0) 219 (66.4) 230 (69.7) 228 (69.1) 248 (75.2) 288 (87.3) 219 (66.4)	123 (37.3) 49 (14.8) 111 (33.6) 91 (27.6) 0 (0.0) 111 (33.6) 100 (30.3) 102 (30.9) 82 (24.8) 42 (12.7) 111 (33.6)
Social status	*P*-value = 0.571		*P*-value = 0.460		*P*-value = 0.23		*P*-value = 0.026^*^		*P*-value = 0.899	
Unmarried Married	1480 (73.0) 1156 (72.1)	547 (27.0) 447 (27.9)	1520 (75.0) 1246 (77.7)	507 (25.0) 357 (22.3)	1907 (94.1) 1536 (95.8)	120 (5.9) 67 (4.2)	1387 (68.4) 1040 (64.9)	640 (31.6) 563 (35.1)	1510 (74.5) 1198 (74.7)	517 (25.5) 405 (25.3)
Occupation	*P*-value = 0.400		*P*-value = 0.931		*P*-value = 0.240		*P*-value = 0.043^*^		*P*-value < 0.001^***^	
High-skilled Low-skilled Skilled (manual) Others	1004 (72.9) 243 (70.8) 68 (80.0) 1321 (72.4)	374 (27.1) 100 (29.2) 17 (20.0) 503 (27.6)	1051 (76.3) 259 (75.5) 67 (78.8) 1385 (75.9)	327 (23.7) 84 (24.5) 18 (21.2) 429 (24.1)	1288 (93.5) 327 (95.3) 83 (97.6) 1745 (95.7)	90 (6.5) 16 (4.7) 2 (2.4) 79 (4.3)	903 (65.5) 235 (68.5) 68 (80.0) 1221 (66.9)	475 (34.5) 108 (31.5) 17 (20.0) 603 (33.1)	1015 (73.7) 282 (82.2) 73 (85.9) 1338 (73.4)	363 (26.3) 61 (17.8) 12 (14.1) 486 (26.6)
Have chronic diseases?	*P*-value = 0.781		*P*-value = 0.512		*P*-value = 0.456		*P*-value < 0.001^***^		*P*-value = 0.013^*^	
No Yes	2119 (72.5) 517 (73.1)	803 (27.5) 190 (26.9)	2220 (76.0) 546 (77.2)	702 (24.0) 161 (22.8)	2767 (94.7) 675 (95.5)	155 (5.3) 32 (4.5)	1898 (65.0) 529 (74.8)	1024 (35.0) 178 (25.2)	2154 (73.7) 554 (78.4)	768 (26.3) 153 (21.6)
Have a previous COVID-19 infection	*P*-value < 0.001^***^		*P*-value = 0.227		*P*-value = 0.116		*P*-value = 0.408		*P*-value = 0.808	
No Yes Not sure	1021 (68.8) 1163 (73.3) 452 (80.7)	463 (31.2) 423 (26.7) 108 (19.3)	1115 (75.1) 1220 (76.9) 421 (75.2)	369 (24.9) 356 (23.1) 139 (24.8)	1395 (94.0) 1510 (95.2) 538 (96.1)	89 (6.0) 76 (4.8) 22 (3.9)	995 (67.0) 1071 (67.5) 361 (64.5)	489 (33.0) 515 (32.5) 199 (35.5)	1107 (74.6) 1189 (75.0) 412 (73.6)	377 (25.4) 397 (25.0) 148 (26.4)
Have family members died from COVID-19?	*P*-value = 0.001^***^		*P*-value = 0.312		*P*-value = 0.384		*P*-value < 0.001^***^		*P*-value = 0.020^*^	
No Yes Maybe	1250 (70.0) 1163 (75.7) 223 (72.4)	535 (30.0) 374 (24.3) 85 (27.6)	1352 (75.7) 1169 (76.1) 244 (79.2)	433 (24.3) 368 (23.9) 64 (20.8)	1696 (95.0) 1460 (95.0) 287 (93.2)	89 (5.0) 77 (5.0) 21 (6.8)	1166 (65.3) 1023 (66.6) 238 (77.3)	619 (34.7) 514 (33.4) 70 (22.7)	1326 (74.3) 1132 (73.6) 250 (81.2)	459 (25.7) 405 (26.4) 58 (18.8)
Continent	*P*-value < 0.001^***^		*P*-value = 0.016^**^		*P*-value = 0.009^**^		*P*-value < 0.001^***^		*P*-value < 0.001^***^	
Asia Africa	1771 (67.1) 865 (87.4)	869 (32.9) 125 (12.6)	1986 (75.2) 781 (78.9)	654 (24.8) 209 (21.1)	2488 (94.2) 955 (96.5)	152 (5.8) 35 (3.5)	1631 (61.8) 796 (80.4)	1009 (38.2) 194 (19.6)	1890 (71.6) 818 (82.6)	750 (28.4) 172 (17.4)

Percentages between brackets

^***^P-value < 0.001, ^**^P-value < 0.01, ^*^P-value < 0.05

### The multilevel logistic model

We found that participants aged between 35 and 50 years had lower odds of having constraints by 44% (adjusted odds ratio (aOR) = 0.56, 95% confidence interval (CI) 0.32–0.99, *p* < 0.05) compared to those in the reference category (18–25 years) (Table 5).

**Table 5:** The results of the multilevel logistic model with random intercept and a random slope for each of the 5C scale, the Middle East and North African countries, 2022 (*N* = 3630)

	Confidence	Complacency	Constraints	Calculation		Collective responsibility	
Predictors	aOR (95% CI)	aOR (95% CI)	aOR (95% CI)	aOR (95% CI)		aOR (95% CI)	
(Intercept)	0.59 (0.24–1.43)	0.61 (0.28–1.30)	0.29^*^ (0.09–0.90)	0.46 (0.16–1.32)		0.30^*^ (0.10–0.88)	
Age categories: 25-<35 years	0.84 (0.66–1.08)	0.91 (0.73–1.15)	0.80 (0.52–1.22)	1.25 (1.00–1.58)		0.99 (0.78–1.27)	
Age categories: 35-< 50 years	0.76 (0.56–1.02)	0.87 (0.66–1.15)	0.56^*^ (0.32–0.99)	1.21 (0.93–1.59)		0.93 (0.70–1.24)	
Age categories: 50–65 years	0.98 (0.67–1.44)	1.01 (0.69–1.48)	0.65 (0.30–1.41)	1.40 (0.98–1.99)		1.23 (0.84–1.78)	
Age categories: above 65 years	1.23 (0.70–2.13)	1.16 (0.67–2.00)	0.41 (0.09–1.86)	0.66 (0.38–1.15)		0.55 (0.29–1.02)	
Gender: male	1.30^**^ (1.09–1.55)	1.22^*^ (1.03–1.45)	1.94^***^ (1.40 – 2.68)	0.80^**^ (0.68–0.95)		0.79^*^ (0.66–0.95)	
Level of education completed: secondary education	0.87 (0.45–1.69)	0.70 (0.36–1.34)	0.38^*^ (0.14–0.97)	1.20 (0.63–2.29)		1.23 (0.60–2.51)	
Level of education completed: university education	0.96 (0.51–1.83)	0.64 (0.34–1.21)	0.26^**^ (0.10 – 0.65)	1.17 (0.62–2.20)		1.20 (0.59–2.41)	
Level of education completed: post-graduated	0.88 (0.45–1.71)	0.49^*^ (0.25–0.95)	0.21^**^ (0.08–0.56)	1.16 (0.61–2.22)		1.33 (0.65–2.73)	
Social status: married	1.09 (0.89–1.34)	1.02 (0.84–1.24)	0.88 (0.60–1.29)	1.07 (0.89–1.28)		1.00 (0.82–1.22)	
Occupation: low-skilled (non-manual)	0.89 (0.65–1.22)	1.02 (0.77–1.37)	0.75 (0.42–1.33)	1.08 (0.82–1.43)		0.81 (0.59–1.11)	
Occupation: skilled (manual)	0.79 (0.43–1.45)	0.64 (0.37–1.12)	0.21^*^ (0.05–0.88)	0.73 (0.41–1.31)		0.71 (0.37–1.37)	
Occupation: others	0.80 (0.64–1.00)	0.79^*^ (0.64–0.98)	0.43^***^ (0.29–0.65)	1.08 (0.88–1.32)		1.14 (0.92–1.41)	
Suffering from chronic diseases	1.05 (0.83–1.32)	1.00 (0.80–1.24)	1.00 (0.65–1.54)	0.81 (0.66–1.01)		1.07 (0.85–1.35)	
Having previous COVID-19 infection (yes)	0.97 (0.69–1.36)	0.90 (0.74–1.08)	0.96 (0.68–1.37)	1.08 (0.91–1.29)		1.06 (0.88–1.28)	
Having COVID-19 infection before (not sure)	0.62^*^ (0.42–0.93)	1.04 (0.82–1.31)	0.68 (0.42–1.12)	1.11 (0.89–1.38)		1.02 (0.81–1.30)	
Having a family members/relatives who died from COVID-19 infection (yes)	1.02 (0.85–1.22)	0.94 (0.79–1.11)	1.14 (0.81–1.59)	1.04 (0.89 – 1.22)		1.17 (0.98–1.38)	
Having family members/relatives who died from COVID-19 infection (not sure)	0.92 (0.67–1.26)	0.87 (0.64–1.19)	1.65 (0.97–2.78)	0.62^**^(0.46–0.83)		0.82 (0.60–1.14)	
Continent: Africa	0.25^**^ (0.09–0.72)	0.81 (0.45–1.45)	0.67 (0.30–1.49)	0.18^*^ (0.04–0.85)		0.23 (0.05–1.10)	
Random effects							
σ^2^_00_		0.60 _Country_	0.17 _Country_	0.26 _Country_	1.30 _Country_		1.25 _Country_
σ^211^		0.21 _Country. had previous COVID-19 infection_					
		0.24 _Country. not sure of having COVID-19 infection_					

*Reference categories:* age: 18-less than 25, Gender: female, Education: primary, Social status: currently unmarried, Occupation: high-skilled (non-manual), Did not have a chronic disease, no previous infection with COVID-19 did not have any of relatives died from COVID-19 infection: Continent: Asia

^*^p < 0.05, ^**^p < 0.01, ^***^p < 0.001

Regarding the gender effect, the odds of confidence, complacency, constraints, performing calculations, and having collective responsibility among men were 1.3 (95% CI 1.09–1.55, *p* < 0.01), 1.22 (95% CI 1.03–1.45, *p* < 0.05), 1.94 (95% CI 1.4–2.68, *p* < 0.001), 0.80 (95% CI 0.68–0.95, *p* < 0.01), and 0.79 (95% CI 0.66–0.95, *p* < 0.05), respectively, compared to among women.

Postgraduate education decreased the odds of complacency (aOR = 0.49; 95%CI: 0.25 – 0.95). Similarly, having completed secondary, university, or postgraduate education decreased the odds of having constraints by [aOR = 0.38, (95% CI 0.14–0.97) *p* < 0.05, (aOR = 0.26, (0.10–0.65), *p* < 0.01, and (aOR = 0.21, (95% CI 0.08–0.56) *p* < 0.01 respectively] compared to the primary education participants. The respondents belonging to the category “others” in the occupation had lower adjusted odds (aOR = 0.79, 95% CI 0.64–0.98, *p* < 0.05).

In addition, being in the categories “skilled manual or others” occupations decreased the odds of having constraints (aOR = 0.21, 95% CI 0.05–0.88, *p* < 0.05, and aOR = 0.43, 95% CI 0.29–0.65, *p* < 0.001, respectively). Participants with a probability of having a previous COVID-19 infection had lower adjusted odds of confidence in COVID-19 (aOR = 0.62, 95% CI 0.42–0.93, *p* < 0.05).

Furthermore, respondents with a probability of having a relative died from COVID-19 infection had lower adjusted odds of performing calculations before taking the COVID-19 vaccine (aOR = 0.62, 95% CI 0.46–0.83, *p* < 0.01). Finally, African respondents had significantly lower adjusted odds of confidence and performed calculations regarding the COVID-19 vaccine compared to Asian respondents (aOR = 0.25, 95% CI 0.09–0.72, *p* < 0.01; aOR = 0.18, 95% CI 0.04–0.85, *p* < 0.05, respectively).

Additionally, the calculated variances of the intercept random effect and slope random effect of the selected variables indicated that the highest variation in the intercept belonged to the calculation model (σ200 = 1.30), followed by the random intercept in the collective responsibility model (σ200 = 1.25). Furthermore, the variances of having previous COVID-19 infection categories ranged between (σ211 = 0.21–0.24) in confidence (Table 5).

The variance of the random effects in the models highlights the degree of variability in each construct across countries, reflecting how much of the outcome variability is attributable to differences between countries.

#### Confidence model

The random effect variance (σ^2^₀ = 0.60) indicates moderate variability in the baseline levels of confidence across countries. This suggests that the unobserved country-specific factors contribute significantly to differences in confidence, beyond the effects explained by the fixed predictors.

#### Complacency model

The random effect variance (σ^2^₀ = 0.17) is relatively small, indicating that the variability in complacency across countries is less pronounced compared to other models. This suggests a more uniform distribution of complacency levels across countries, with less country-specific variance influencing the outcomes. The additional variance associated with countries having had previous COVID-19 infections (σ^2^₁₁ = 0.21) suggests that this factor also introduces some variability in confidence, although to a lesser extent. This implies that while the general level of confidence varies across countries, the influence of prior COVID-19 infections further modifies this variability, albeit not as strongly. Moreover, the variance associated with uncertainty about previous COVID-19 infections (σ^2^₁₁ = 0.24) also contributes to this model, indicating some level of country-specific variability.

#### Constraints model

The random effect variance (σ^2^₀ = 0.26) reveals a moderate level of variability in constraints experienced across different countries. This variance suggests that constraints are perceived differently depending on country-specific factors, which are not fully accounted for by the fixed effects in the model.

#### Calculation model

The variance of the random effect (σ^2^₀ = 1.30) is the highest among the models, reflecting substantial variability in the perception of calculation-related issues across countries. This high variance indicates that countries experience and interpret calculation-related factors quite differently, suggesting a strong influence of country-specific factors on this outcome. This variability could be attributed to diverse contextual factors that affect how calculation issues are perceived and managed in different settings.

#### Collective responsibility model

Similarly, the random effect variance (σ^2^₀ = 1.25) is also high, indicating significant variability in the perception of collective responsibility across countries. This suggests that the concept of collective responsibility is substantially influenced by country-specific differences, which are not fully captured by the fixed effects. The considerable variance in this model reflects the impact of cultural, social, or other contextual factors on how collective responsibility is understood and acted upon in various countries (Table 5).

### The goodness-of-fit measures

The goodness of fit of the model was assessed using various criteria. The three measures (The Akaike Information Criterion (AIC), the Bayesian Information Criterion (BIC), and Likelihood ratio test) were used to evaluate the transition from the random intercept model to the random intercept and slope models. The AIC and deviance were lower in the model with an added random slope. However, the BIC was slightly higher in the four models owing to the increased complexity. When the random slope was added to the confidence model, the AIC decreased from 3726.1 to 3722.1. Furthermore, the likelihood ratio test was significant in all four models when a random slope was added to the model (*p* < 0.05) (Supplementary material S2: Table S1].

In the current study, the psychological antecedents of 5C were significantly correlated with the acceptance of the COVID-19 vaccine. A significant positive correlation was found between vaccine confidence and administration of at least one vaccine dose. Increasing confidence increased acceptance of the vaccine (Correlation coefficient (*r*) = 0.31, *p* < 0.01). In addition, there was a significant positive correlation between collective responsibility and the administration of at least one vaccination dose. Increasing collective responsibility increased vaccine acceptance (*r* = 0.18, *p* < 0.01) (Table 6).

**Table 6:** Point-biserial correlation coefficient between psychological antecedents of 5c and COVID-19 vaccination, the Middle East and North African countries, 2022 (*N* = 3630)

Variables	Confidence domain	Complacency domain	Constraints domain	Calculation domain	Collective responsibility domain
Intention to take the	0.31^**^	− 0.09^*^^*^	− 0.07^*^^*^	− 0.07^*^^*^	0.18^*^^*^
COVID-19 vaccination	< 0.01	< 0.01	< 0.01	< 0.01	< 0.01

^**^p < 0.01

The other three psychological antecedents of 5c were found to have a significant negative correlation with the intake of the COVID-19 vaccine. Increasing complacency would decrease vaccine acceptance (*r* = − 0.09, *p* < 0.01). Increasing the number of calculations would decrease acceptance of the vaccine (*r* = − 0.07, *p* < 0.01). High constraints would decrease vaccine acceptance (*r* = − 0.07, *p* < 0.01) (Table 6).

## Discussion

Many countries in the MENA, such as Libya, Sudan, Yemen, and Syria, still have low rates of COVID-19 vaccination coverage after approximately 3 years of vaccine availability. Although vaccines are cost-free and obligatory in many countries, factors other than individual and country income have resulted in vaccine inequity in these countries [18, 29]. Therefore, we attempted to discover the determinants of the psychological antecedents in the 11 countries under consideration and unravel the psychological factors that push individuals to take or refuse the vaccine after its actual distribution.

After applying a multilevel logistic regression model, considering the hierarchical data structure, the researchers found that gender affects all domains of the 5C scale, as males had significantly higher confidence, complacency, and constraints than females and had significantly lower calculation and collective responsibility than females. A systematic review and meta-analysis study documented that the majority of research found that men had increased intentions to get vaccinated against COVID-19. The obligation of vaccination in government and workplace settings and travel restrictions without proof of COVID-19 vaccination may influence males to be vaccinated. Females consider the protection of their families more seriously. This may explain the higher collective responsibility of women compared to men [30].

Approximately three-quarters of the respondents under the age of 50 years had no confidence in COVID-19 vaccinations. Women were less confident than men, and this result persisted after performing a regression model. Respondents who were less confident in the safety and efficacy of the vaccine tended to have a lower rate of acceptance of the vaccine. Similar to the previous results, Qunaibi et al. (2021) stated that Arab women and middle-aged people had negative associations with vaccine acceptance [31]. Young age, low confidence in the COVID-19 vaccine and the health care authorities during the pandemic, and inadequate information provided by the health authorities were the main factors enhancing the rejection of vaccination [32].

The current study revealed that the higher the education level, the lower the percentage of respondents who were confident in the COVID-19 vaccination. Similar results were reported by a study conducted in Turkey and the United Kingdom. It documented that participants with higher educational levels had lower acceptance of and confidence in the COVID-19 vaccine [33].

Additionally, the present study found that participants with a probability of having a previous COVID-19 infection had lower confidence in the COVID-19 vaccine than those who had not been previously infected. After studying many low- and middle-income countries, Solís Arce et al. [34] revealed that the main cause of vaccine acceptance was the hope of people to protect themselves from COVID-19 infection. In contrast, the fear of side effects of vaccines was the major cause of vaccine rejection. This may explain our finding, as participants who had already been infected felt no need to be vaccinated after infection.

The current study showed that respondents from Afghanistan had the highest confidence in the vaccine. However, African respondents were generally less confident than Asian respondents. Similarly, a previous cross-sectional study among Arab healthcare workers documented that the lowest confidence in the vaccine was among participants from African countries in the Arab world (Egypt, Morocco, Tunisia, and Algeria) due to a lack of trust in vaccines or fear of their side effects [35].

Trust in vaccine safety is one of the most important factors affecting the intention to be vaccinated. Confidence in the government and healthcare system is essential to increase vaccine acceptance. The need to rebuild trust in vaccines and health authorities is mandatory to increase vaccine uptake and equity [15]. Parents had a higher acceptance of vaccinating their children when they trusted the vaccine. In addition, a higher perception of the benefits of vaccination was associated with higher acceptance of vaccination by participants and their children [36]. Misinformation about the COVID-19 vaccination and its probable health risks reduces public confidence in the vaccine. Therefore, removing these misconceptions through effective mass media messages is important for increasing vaccine uptake and improving vaccine equity.

In the current study, participants with postgraduate education had a lower complacency than those with primary education. Additionally, respondents from Morocco had the highest complacency among all the countries. Similarly, a previous study reported a high level of complacency in Morocco and Jordan [29]. If a person believes that their immune system is strong enough to protect them from infection, they will not receive the vaccine. Complacent Chinese individuals do not aim to receive vaccination [37].

Respondents aged 35–50 years had lower constraints than those aged 18–25 years. The lower the educational level, the higher the percentage of constraints. According to occupation, skilled manual participants had lower constraints than high-skilled non-manual participants. Pakistan and Saudi Arabia had the highest constraints, as recorded in the present research. Countries with conflicts during the pandemic, such as Lebanon, Syria, Iraq, and Palestine, had high rates of morbidity and mortality from COVID-19, as the surrounding environment would favor the spread of the infection and hinder the accessibility to vaccines in addition to the compromised health care system in these countries [38]. A cross-sectional Iraqi study in 2023 reported that vaccine hesitancy was related to increased vaccination barriers. Perceived constraints may be due to a lack of accessibility in service delivery [39].

Calculation means weighing the benefits versus risks of vaccination before taking action towards vaccination. The present study revealed that participants with a probability of having a deceased relative due to COVID-19 had lower calculations before receiving the COVID-19 vaccine than those without a deceased relative to the disease. Approximately one-third of the respondents under the age of 65 performed the calculations before vaccination. Females performed calculations more frequently than males. Individuals with chronic diseases performed fewer calculations than those without. Asian countries performed more calculations than African countries. In the current study, respondents from Iraq and Yemen showed higher vaccination calculations. For Yemen, the risk calculation could be high because of the current conflict with the low number of reported cases and deaths. Poor health services may be another contributing factor. The low fatality rate (approximately 1%) in Iraq could also be an explanation. A cross-sectional study reported that Sudan and Egypt had the highest calculations when their vaccination coverage was very low (1.3% and 8.1%, respectively). A high calculation can lower vaccination acceptance and, hence, affect vaccine equity. People search for more information about vaccination on the Internet when they do not trust local authorities’ information. The Internet and social media platforms have misleading and faulty information about COVID-19 vaccines, which may lead to the avoidance of vaccination [29].

Females had more collective responsibilities than males. Respondents from Egypt, Iraq, and Pakistan showed greater collective responsibility than those from other countries. Similar to our results, a qualitative study investigated factors affecting the intention to vaccinate with COVID-19 among the Arab population and found increased collective responsibility among participants as they were committed to safeguarding others through vaccination. Increasing awareness about the risk of COVID-19 in one’s social circle could increase vaccination acceptance through increased collective responsibility [15]. The desire of a person to protect other people from being infected by becoming vaccinated could be a strong motivation to get the vaccine and thus enhance vaccine equity. This motivation might be even stronger for women. This could be because women tend to express greater concern for the well-being of those around them. Vaccination efforts could be more effective if the risks to people close to the participants are proven. Furthermore, vaccination is a valuable method to explain the concept of herd immunity [40].

The psychological antecedents of vaccination are crucial to understanding vaccine equity. The present study found that increasing confidence in vaccines and collective responsibility towards relatives and the community was related to increasing acceptance of the COVID-19 vaccine. The reduction in complacency, calculations, and constraints was found to be associated with acceptance of the COVID-19 vaccine. The present study is novel in examining the psychological antecedents as hidden factors to explain the vaccine inequity in the MENA region. Similar results were documented by studying the psychological antecedents of the COVID-19 vaccine among black Americans. Confidence and collective responsibility were directly predicting vaccination intentions, while confidence, calculation, and collective responsibility were significantly expected attitudes toward COVID-19 vaccination [41]. Vaccine inequity is powered by psychological factors beyond just affordability. Fear of side effects, distrust in medical authorities due to past experiences or misinformation, and hesitancy to get vaccinated can all contribute. These factors can be particularly prevalent in low-income countries, further hindering access and creating a cycle of lower vaccination rates and higher health risks [42].

Researchers have used various statistical models to predict how many people in a population might get sick or die from COVID-19 and how widespread vaccination can reduce these numbers. The wider the distribution of vaccines, the less likely people are to suffer health problems from the virus. Their findings suggested that prompt access to vaccines in low- and middle-income countries could have significantly reduced deaths, with a potential range of 6% to over 50% fewer fatalities. These results highlight the importance of equitable vaccine distribution not only for public health but also for minimizing the overall burden on healthcare systems. This, in turn, translates to a lighter economic burden from COVID-19 on society [43, 44].

### Limitations and strengths

The current study has some limitations. First, collecting data through cross-sectional online surveys may lead to sampling bias that may limit the representativeness of the results. Second, self-reported data could be skewed by recall bias and a tendency to present socially desirable responses. Third, the study respondents participated through a non-random sampling method, which may have affected the generalizability of the study. Fourth, conducting the survey online may exclude individuals without internet access or those not technologically proficient, potentially skewing the sample towards younger, more educated, and more affluent individuals.

Despite these limitations, the study has many points of strength. First, we detected 5C as an indirect determinant of vaccination coverage. We used 5C in another area (to explain the present situation), rather than its primary use (to predict vaccine intention). Second, we evaluated the relationship between vaccination inequity and the psychological factors that lead to the acceptance or rejection of vaccines, which has rarely been studied in the MENA region. Third, we focused on actual vaccination behavior after the vaccine was rolled out, rather than vaccine intention only. Fourth, the statistical analysis was performed using multilevel analysis, allowing us to view the differences in outcomes across various countries. A multilevel logistic regression model was used to capture the clustering of observations in each country and estimate the explanatory variables’ effects on each item of the 5Cs of psychological factors in 11 countries in the MENA region. Nonetheless, the findings suggest a significant likelihood test between the random intercept model and the random intercept and slope model, suggesting variability in the impact of certain factors across different countries (such as having previous COVID-19 infection in the “Confidence Model”). This indicates the variability between different countries in the log of the odds of the 5Cs. Furthermore, the variability in the five outcome variables among the 11 countries was 22%, 5%, 10%, 38%, and 33% of the total variability, respectively. Additionally, the variability in 5C between the study countries confirmed the various effects of psychological antecedents on vaccination coverage, stimulating vaccination inequity between them. Based on all the previous points, we believe that this study could provide policymakers and public health managers with valuable information on the key determinants of the MENA population regarding COVID-19 vaccination, helping them to improve the acceptance rate of any new vaccine by targeting the psychological drivers of the people.

## Conclusions

This research is novel in using the 5C scale to explore the vaccine inequity in the MENA region by using a multilevel logistic regression model for COVID-19 vaccination intention. The 5C domains were influenced by gender, country, and continent. After applying a multilevel logistic regression model, the researchers found that gender affected all the domains of the 5C scale. Age, education, and occupation have an impact on the constraint domain. Education level affects complacency. Previous COVID-19 infection changed the degree of confidence. Having a dead relative due to COVID-19 lowered the calculations. Being African or Asian changed confidence and calculations regarding the COVID-19 vaccination. People with higher confidence and collective responsibility were more likely to get vaccinated. Conversely, increasing complacency, calculations, and constraints were associated with lower vaccine acceptance.

To improve vaccination rates and address vaccine inequity, it's crucial to understand the specific needs and beliefs of the target population. Designing policies and programs that consider social and psychological factors is essential. Making vaccination services accessible and building trust in vaccines are also key strategies. By addressing these factors, we can improve public health and protect communities from infectious diseases like COVID-19. Future research on vaccine inequity should explore multidimensional solutions, including psychological, social, and cultural factors, logistical challenges, and innovative financing models.

## Supplementary Information

Supplementary Material 1.

Supplementary Material 2.

Supplementary Material 3.

## Data Availability

The datasets used and/or analyzed during the current study are available from the corresponding author on reasonable request.

## Abbreviations

5Cs: Psychological factors: confidence, complacency, constraints, calculation, and collective responsibility
σ^2^: Variances
*χ*2: Chi-square test
β: Regression coefficient
AIC: Akaike information criterion
aOR: Adjusted odds ratio
BIC: Bayesian information criterion
CDC: Centers for Disease Control and Prevention
CI: Confidence interval
COVID–19: Coronavirus disease 2019
ICC: Intraclass correlation
LRT: Likelihood Ratio Test
MENA: Middle East and North African Countries
No.: Number
OR: Odds ratio
*P*: *P-*Value
*r*: Correlation coefficient
UAE: United Arab Emirates
VIF: Variance inflation factor
WHO: World Health Organization

## Footnotes

1 If the ICC is small, this means that the correlation inside the same country may be small and we might not need the random intercept, but it does not assure the non-presence of the random effect of the slope (26).

2 We checked for multicollinearity between variables, and all the variables had variance inflation factor (VIF) < 4.
