# Supplementary material for: Psychological antecedents of vaccine inequity: keys to improve the rates of vaccination
Source: J Egypt Public Health Assoc. 2024 Dec 4;99:31. doi: 10.1186/s42506-024-00175-7 (PMC11615162; doi:10.1186/s42506-024-00175-7)
Supplement: Supplementary file 1 — Supplementary Material 1. [file 42506_2024_175_MOESM1_ESM.docx]

**Supp. Material S1 Questionnaire**

**Psychological Antecedents of Vaccine Inequity: Keys to Improve the Rates of Vaccination**

**Mohamed Fakhry Hussein ^1*^, Sarah Assem Ibrahim^2^, Suzan Abdel-Rahman^2^, Abdelhamid Elshabrawy ^2^, Haqema Ahmed Abduh Nasr ^3^, Saja Yazbek ^4^, Abdul Jabbar ^5^, Cinaria Tarik Albadri ^6^, Mariam Alsanafi ^7^, Narjiss aji ^8^, Naglaa Youssef ^9^, Hammad Mohammad Hammad ^10^, Fatimah Saed Alabd Abdullah ^11^, Ehab Elrewany ^1^, Mohamed Mostafa Tahoun ^1^, Mahmoud Tolba ^12^, Mohamed Khaled Abo Salama ^13^, Ramy Mohamed Ghazy ^1^**

This research aims to estimate the psychological antecedents of accepting the COVID-19 vaccination. All your information will be confidential and no names will be used when publishing the search. The study was approved by The Ethics Committee of the Faculty of Medicine, Alexandria University, Egypt (IRB number: 00012098). The duration to complete the questionnaire is 5- 10 minutes. You are absolutely free not to participate in the research at any time. Thank you for sharing your valuable time with us. If you agree to register in the search, press Continue.

For any inquiry please contact Dr. Mohamed Fakhry Hussein email: [hiph-mohamedfakhry@alexu.edu.eg](mailto:hiph-mohamedfakhry@alexu.edu.eg)

**I accept to be involved in the research**

- Continue
- Do not want to be in the research

**Chose the language**

- العربية
- English

1. **Respondent socio-demographic data**
2. **Age**

- Less than 18 years
- 18 - less than 25 years
- 25 - less than 35 years
- 35 - less than 50 years
- 50 - 65 years
- Above 65 years

1. **Gender:**

- Male
- Female

1. **Level of education completed**

- Primary education
- Secondary education
- University education
- Post graduated

1. **Marital status**

- Married
- Single
- Widow
- Divorced

1. **Occupation**

- Manager
- Professional job as in medical field or engineer or chemist
- Technicians and Associate Professionals
- Clerical Support Workers
- Service and Sales Workers
- Skilled Agricultural, Forestry and Fishery Workers
- Craft and Related Trades Workers
- Plant and Machine Operators, and Assemblers
- Elementary Occupations
- Armed Forces Occupations
- Student
- Not working / Retired

1. **Respondent history of chronic diseases and COVID-19 infection**
2. **Do you suffer from chronic diseases?**

- Yes
- No

1. **Have you had COVID-19 infection before?**

- Yes
- No
- I do not know

1. **Do you have any family members/ relatives who died of COVID-19 infection?**

- Yes
- No
- Maybe

**III. Respondent psychological antecedents towards COVID-19**

**Please answer these questions about the COVID-19 vaccines**

1. **I am completely confident that vaccines are safe.**

Strongly Disagree

- 1
- 2
- 3
- 4
- 5
- 6
- 7

Strongly agree

1. **Vaccinations are effective.**

Strongly Disagree

- 1
- 2
- 3
- 4
- 5
- 6
- 7

Strongly agree

1. **Regarding vaccines, I am confident that public authorities decide in the best interest of the community.**

Strongly Disagree

- 1
- 2
- 3
- 4
- 5
- 6
- 7

Strongly agree

1. **Vaccination is unnecessary because vaccine-preventable diseases are not common anymore.**

Strongly Disagree

- 1
- 2
- 3
- 4
- 5
- 6
- 7

Strongly agree

1. **My immune system is so strong, it also protects me against diseases.**

Strongly Disagree

- 1
- 2
- 3
- 4
- 5
- 6
- 7

Strongly agree

1. **Vaccine-preventable diseases are not so severe that I should get vaccinated.**

Strongly Disagree

- 1
- 2
- 3
- 4
- 5
- 6
- 7

Strongly agree

1. **Everyday stress prevents me from getting vaccinated.**

Strongly Disagree

- 1
- 2
- 3
- 4
- 5
- 6
- 7

Strongly agree

1. **For me, it is inconvenient to receive vaccinations.**

Strongly Disagree

- 1
- 2
- 3
- 4
- 5
- 6
- 7

Strongly agree

1. **Visiting the doctor's makes me feel uncomfortable; this keeps me from getting vaccinated.**

Strongly Disagree

- 1
- 2
- 3
- 4
- 5
- 6
- 7

Strongly agree

1. **When I think about getting vaccinated, I weigh benefits and risks to make the best decision possible.**

Strongly Disagree

- 1
- 2
- 3
- 4
- 5
- 6
- 7

Strongly agree

1. **For each and every vaccination, I closely consider whether it is useful for me**

Strongly Disagree

- 1
- 2
- 3
- 4
- 5
- 6
- 7

Strongly agree

1. **It is important for me to fully understand the topic of vaccination, before I get vaccinated.**

Strongly Disagree

- 1
- 2
- 3
- 4
- 5
- 6
- 7

Strongly agree

1. **When everyone is vaccinated, I don’t have to get vaccinated, too.**

Strongly Disagree

- 1
- 2
- 3
- 4
- 5
- 6
- 7

Strongly agree

1. **I get vaccinated because I can also protect people with a weaker immune system.**

Strongly Disagree

- 1
- 2
- 3
- 4
- 5
- 6
- 7

Strongly agree

1. **Vaccination is a collective action to prevent the spread of diseases.**

Strongly Disagree

- 1
- 2
- 3
- 4
- 5
- 6
- 7

Strongly agree

Instruction: “Please evaluate how much you disagree or agree with the following statements.” (1 = strongly disagree, 2 = moderately disagree, 3 = slightly disagree, 4 = neutral, 5 = slightly agree, 6 = moderately agree, 7 = strongly agree).

**Thanks a lot for your time**
