# Supplementary material for: Psychological antecedents of vaccine inequity: keys to improve the rates of vaccination
Source: J Egypt Public Health Assoc. 2024 Dec 4;99:31. doi: 10.1186/s42506-024-00175-7 (PMC11615162; doi:10.1186/s42506-024-00175-7)
Supplement: Supplementary file 2 — Supplementary Material 2. [file 42506_2024_175_MOESM2_ESM.docx]

**Supp. Material S2 Figures**

**Psychological Antecedents of Vaccine Inequity: Keys to Improve the Rates of Vaccination**

**Mohamed Fakhry Hussein ^1*^, Sarah Assem Ibrahim^2^, Suzan Abdel-Rahman^2^, Abdelhamid Elshabrawy ^2^, Haqema Ahmed Abduh Nasr ^3^, Saja Yazbek ^4^, Abdul Jabbar ^5^, Cinaria Tarik Albadri ^6^, Mariam Alsanafi ^7^, Narjiss aji ^8^, Naglaa Youssef ^9^, Hammad Mohammad Hammad ^10^, Fatimah Saed Alabd Abdullah ^11^, Ehab Elrewany ^1^, Mohamed Mostafa Tahoun ^1^, Mahmoud Tolba ^12^, Mohamed Khaled Abo Salama ^13^, Ramy Mohamed Ghazy ^1^**

**
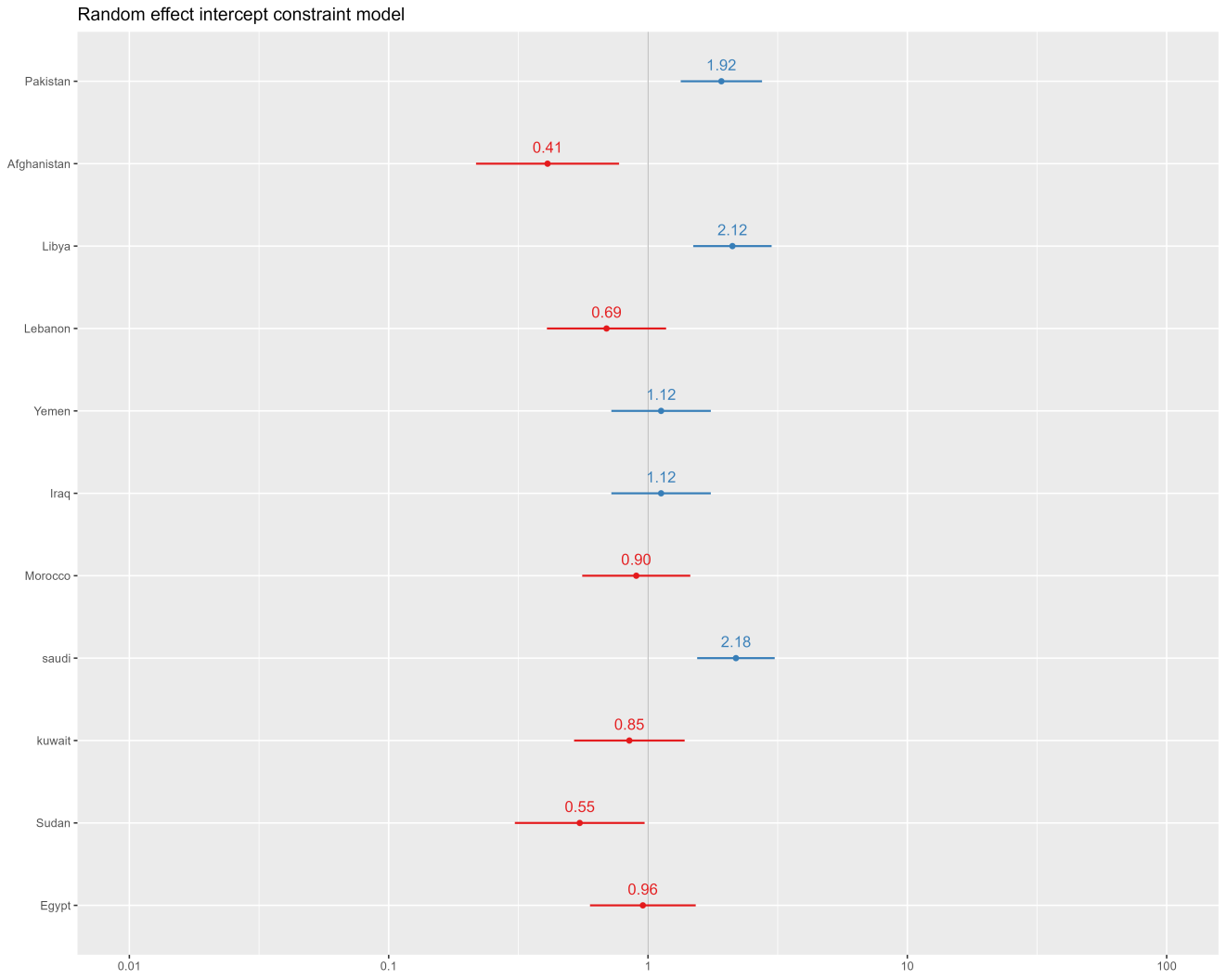
**

**Fig A.1:** Random effect intercept for constraint model, the Middle East and North Africa countries, 2022 (No.=3630)

**
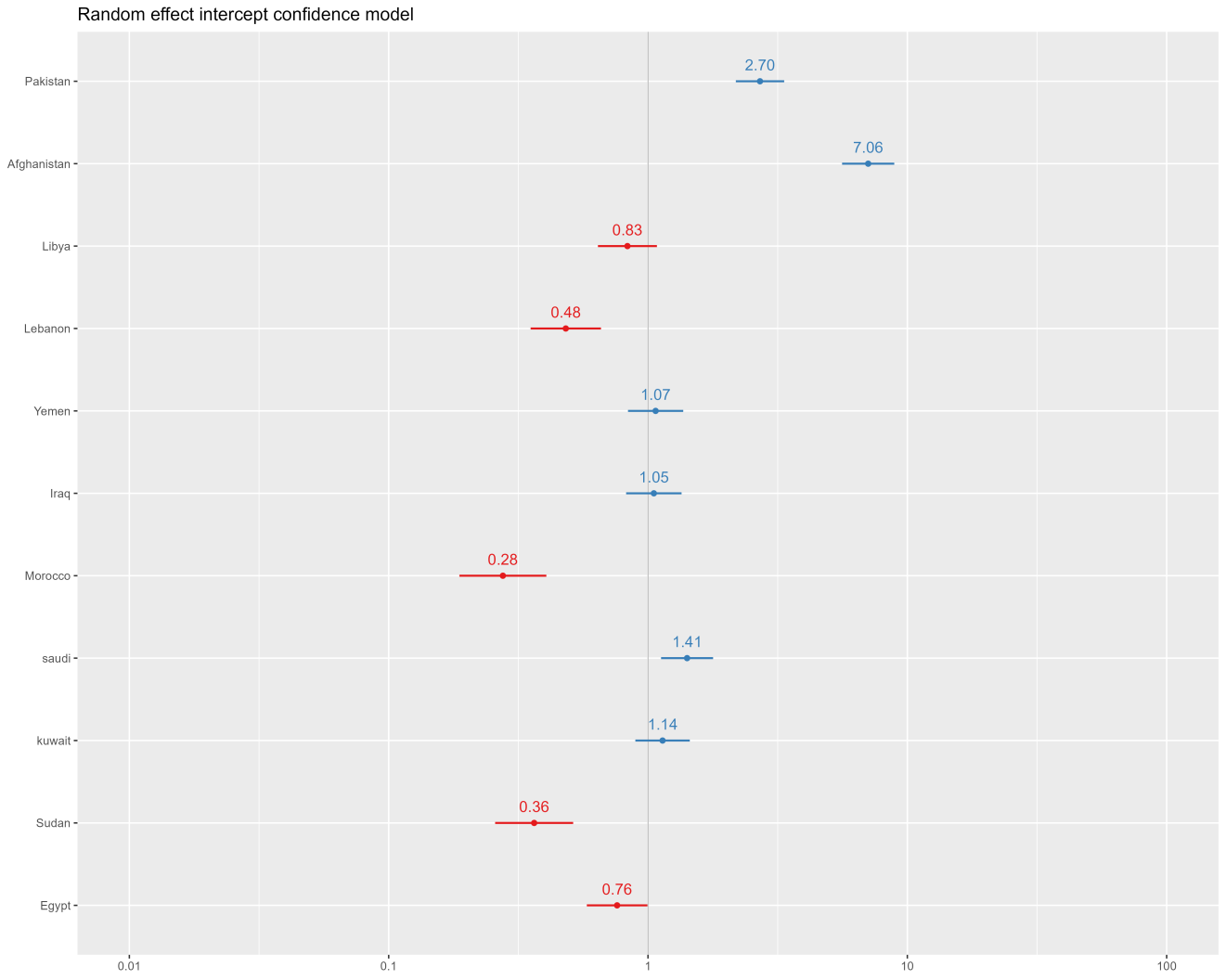
Fig A.2:** Random effect intercept for confidence model, the Middle East and North Africa countries, 2022 (No.=3630)

**
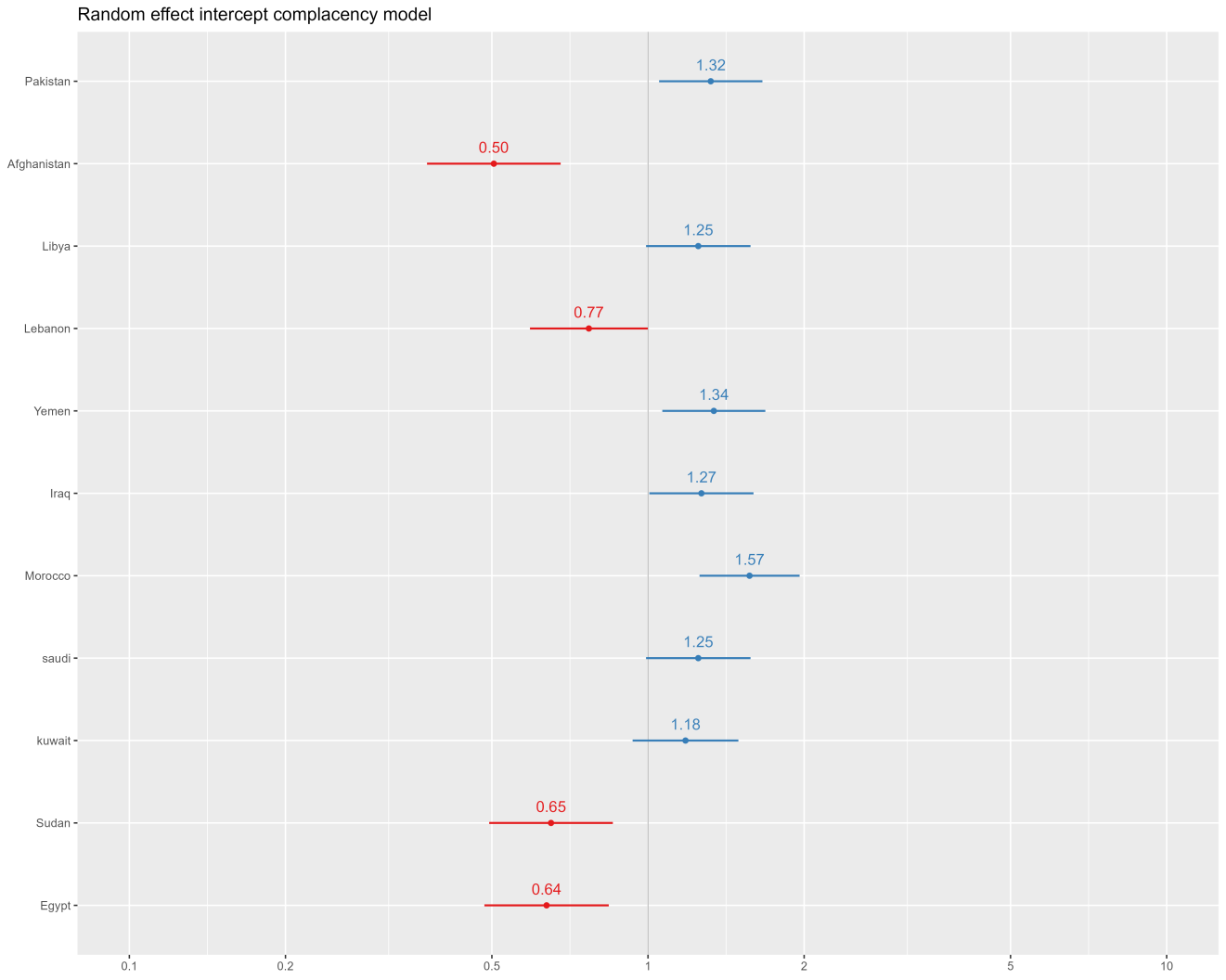
**

**Fig A.3:** Random effect intercept for complacency model, the Middle East and North Africa countries, 2022 (No.=3630)

**
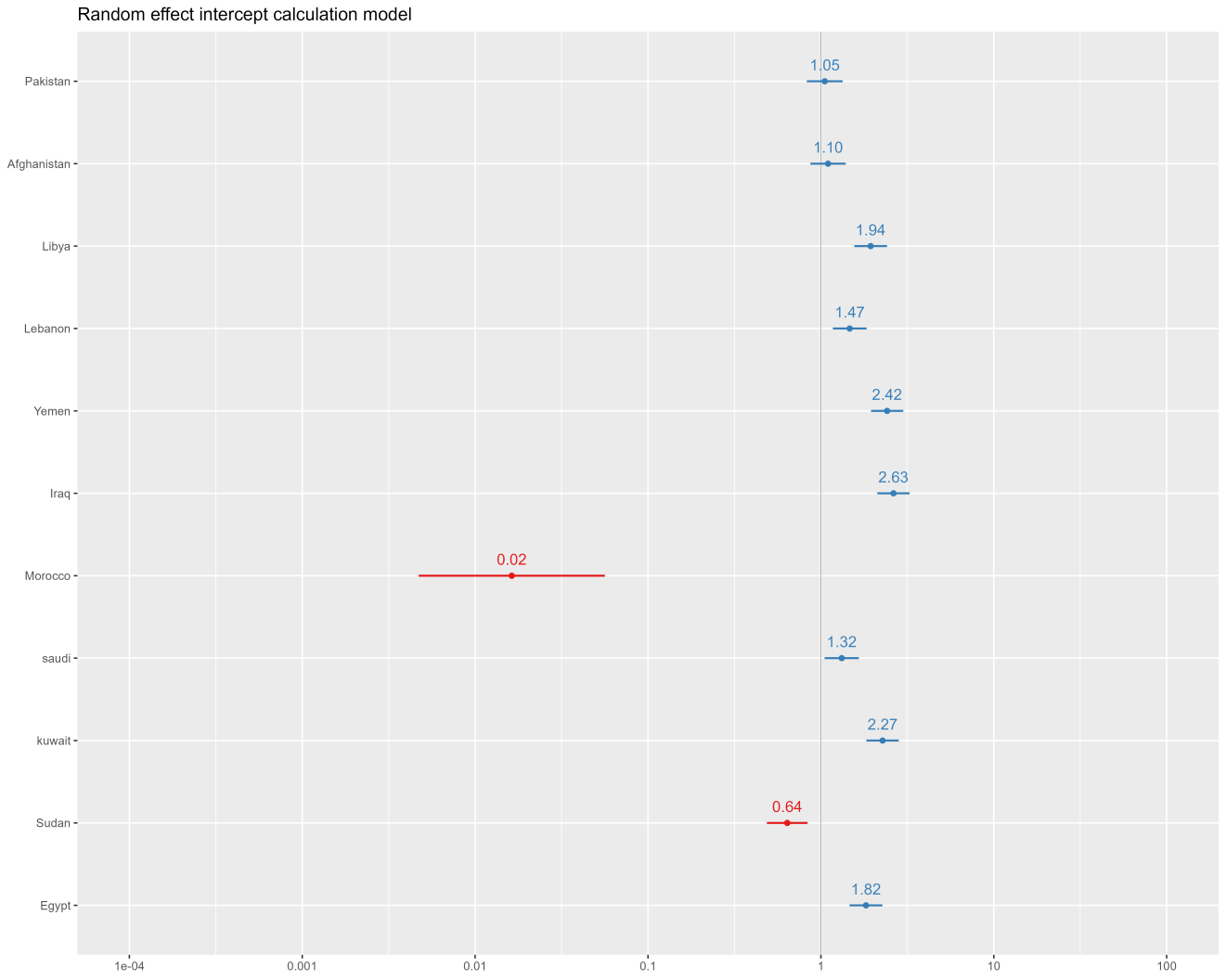
**

**Fig A.4:** Random effect intercept for calculation model, the Middle East and North Africa countries, 2022 (No.=3630)

**
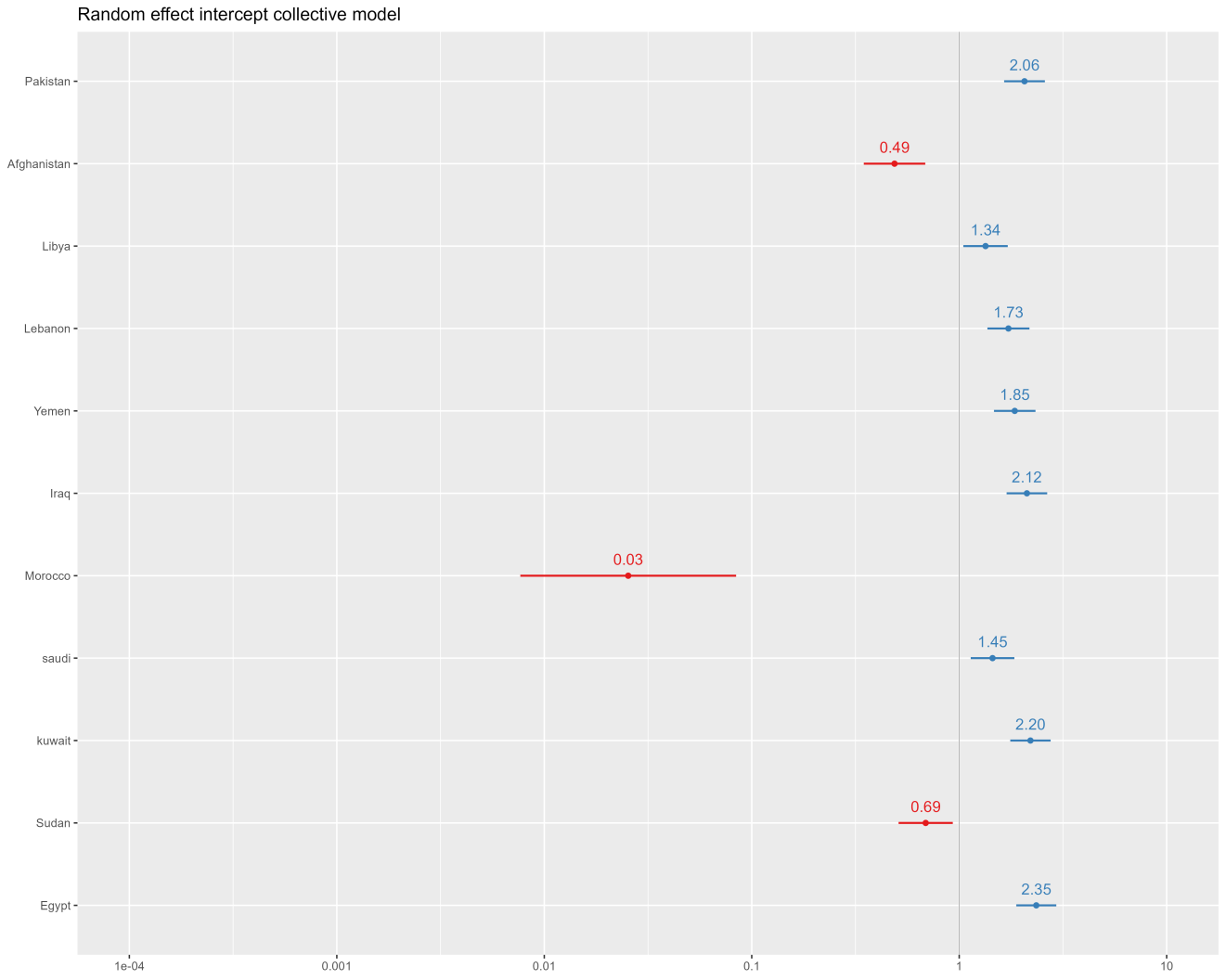
**

**Fig A.5:** Random effect intercept for collective responsibility model, the Middle East and North Africa countries, 2022 (No.=3630)

**Model Fit Indices**

The Table S1 presents Model Fit Indices for the confidence model, comparing two competing models: one with a random intercept and another with both a random intercept and a random slope. The indices provided include the Akaike Information Criterion (AIC), Bayesian Information Criterion (BIC), and deviance, which are widely used to evaluate the goodness of fit in statistical models.

The AIC for the random intercept model is 3726.1, whereas it decreases to 3722.1 when a random slope is introduced. Since a lower AIC value indicates a better model fit, the decrease suggests that the random intercept and slope model offers an improvement in fit over the simpler random intercept model. However, the difference between the two values is relatively small, indicating only a marginal improvement in fit when the random slope is added.

Likewise, the Deviance, which measures the model's lack of fit, decreases from 3686.1 in the random intercept model to 3672.1 in the random intercept and slope model. A lower deviance reflects a better fit, aligning with the AIC in suggesting that the model with a random slope fits the data better than the random intercept model alone.

In contrast to the AIC, the BIC penalizes model complexity more heavily. The BIC for the random intercept model is 3850, but it increases to 3877 when the random slope is included. This increase in BIC suggests that although the model with the random slope fits the data better, the added complexity of this model might not be fully justified. BIC values favor models that balance goodness of fit with simplicity, and the higher BIC in the random slope model indicates a preference for the simpler random intercept model under this criterion.

The results demonstrate a trade-off between model fit and complexity. While AIC and deviance both indicate that the random intercept and slope model provides a better fit, the increase in BIC suggests that the added complexity may not be warranted. Thus, the decision to favor one model over the other depends on the specific context of the research and the balance between improving model fit and maintaining model simplicity.

The likelihood ratio test yielded significant results (p < 0.0001) in the confidence model with the random slope for the previous infection variable, supporting the inclusion of the random slope. This indicates that the random slope model provides a statistically significant improvement in fit compared to the random intercept-only model, despite the increase in BIC, which penalizes for additional parameters.

**Table S1.** Selection criteria for multilevel logistic regression models, the Middle East and North Africa countries, 2022 (No.=3630)

| **Criteria (confidence model)** | **Model with random intercept** | **Model with random intercept and random slope** |
| --- | --- | --- |
| **AIC** | 3726.1 | 3722.1 |
| **BIC** | 3850 | 3877 |
| **Deviance** | 3686.1 | 3672.1 |
